# Virtual pathology of cervical radiculopathy based on 3D MR/CT fusion images: impingement, flattening or twisted condition of the compressed nerve root in three cases

**DOI:** 10.1186/s40064-015-0898-6

**Published:** 2015-03-12

**Authors:** Junji Kamogawa, Osamu Kato, Tatsunori Morizane, Taizo Hato

**Affiliations:** Spine & Sports Center, Shiraishi Hospital, 1-5-9 Matsumoto Town, Imabari City, Ehime 794-0041 Japan; Department of Radiology, Shiraishi Hospital, 1-5-9 Matsumoto Town, Imabari City, Ehime 794-0041 Japan

**Keywords:** 3D MR/CT fusion image, Cervical radiculopathy, Nerve root, Herniated disc, Bony spur, Twisted condition, Pathoimagiology, Microscopic surgery

## Abstract

**Background:**

There have been several imaging studies of cervical radiculopathy, but no three-dimensional (3D) images have shown the path, position, and pathological changes of the cervical nerve roots and spinal root ganglion relative to the cervical bony structure. The objective of this study was to introduce a technique that enables the virtual pathology of the nerve root to be assessed using 3D magnetic resonance (MR)/computed tomography (CT) fusion images that show the compression of the proximal portion of the cervical nerve root by both the herniated disc and the preforaminal or foraminal bony spur in patients with cervical radiculopathy.

**Findings:**

MR and CT images were obtained from three patients with cervical radiculopathy. 3D MR images were placed onto 3D CT images using a computer workstation.

The entire nerve root could be visualized in 3D with or without the vertebrae. The most important characteristic evident on the images was flattening of the nerve root by a bony spur. The affected root was constricted at a pre-ganglion site. In cases of severe deformity, the flattened portion of the root seemed to change the angle of its path, resulting in twisted condition.

**Conclusions:**

The 3D MR/CT fusion imaging technique enhances visualization of pathoanatomy in cervical hidden area that is composed of the root and intervertebral foramen. This technique provides two distinct advantages for diagnosis of cervical radiculopathy. First, the isolation of individual vertebra clarifies the deformities of the whole root groove, including both the uncinate process and superior articular process in the cervical spine. Second, the tortuous or twisted condition of a compressed root can be visualized.

The surgeon can identify the narrowest face of the root if they view the MR/CT fusion image from the posterolateral-inferior direction. Surgeons use MR/CT fusion images as a pre-operative map and for intraoperative navigation. The MR/CT fusion images can also be used as educational materials for all hospital staff and for patients and patients’ families who provide informed consent for treatments.

## Introduction

Cervical radiculopathy is a common spinal disorder in adults. It is caused by compression of the cervical nerve root and is most commonly reported for the C7 nerve (60% of cases) and C6 nerve (25% of cases) (Murphey et al. 1973; Radhakrishnan et al. 1994). Typical clinical symptoms are neck and shoulder pain, radiating pain along the distribution of the nerve root, paresthesia, diminished sensation to pin prick, diminished reflexes, muscle weakness, and, rarely, muscle wasting in the neck and ipsilateral upper extremity (Cave et al. 1955; Boyce et al. 2003; Kim et al. 2010). Foraminal encroachment of the spinal nerve root due to degenerative changes in the uncovertebral and zygapophyseal joints and herniation of the nucleus pulposus are the two most common causes of cervical radiculopathy (Carette et al. 2005). Although 40–80% of patients with cervical radiculopathy respond well to conservative treatment (Lees et al. 1963; Honet et al. 1976; Gore et al. 1987; Kim et al. 2010; Thoomes et al. 2012), some patients experience persistent radicular pain and progressive muscle weakness after conservative treatment and require surgery. The effectiveness of posterior foraminotomy for cervical radiculopathy is over 80% (Jödicke et al. 2003; Korinth et al. 2006). There are no published guidelines by professional societies for the assessment and management of cervical radiculopathy (Carette et al. 2005; Kim et al. 2010; Bono et al. 2011; Thoomes et al. 2012).

The major problem in diagnosing degenerative cervical radiculopathy is that there is no imaging modality that provides detailed three-dimensional (3D) images of the cervical nerve roots. Magnetic resonance (MR) imaging is the approach of choice in patients with cervical radiculopathy, but there are no clear guidelines for nerve root detection and MR imaging of the root differs across hospitals. Computed tomography (CT) alone is of limited value in assessing cervical radiculopathy, although it is useful in distinguishing the extent of a bony spur or foraminal encroachment or the presence of ossification of the posterior longitudinal ligament. Even CT myelography cannot accurately detect the whole nerve root. Most spinal surgeons are able to mentally combine CT and MR images to perform an accurate assessment of the pathoanatomical structure. Evaluation of cervical radiculopathy requires both imaging modalities because the nerve root is a very small and soft organ, whereas the bony spur is very hard. The site of the affected nerve root and the decompressed amount of bony spur both must be considered when planning surgery.

Although there have been several studies of cervical imaging (Scotti et al. 1983; Modic et al. 1986; Teresi et al. 1987; Wilson et al. 1991; Houser et al. 1993; Ilkko et al. 1996), few have identified delicate nerve compression near or in the intervertebral foramen and none have shown the compressed cervical nerve root and bony spur in a single, 3D image that combines CT and MR images. In 2009, we reported the first 3D MR/CT fusion images of the cranio-vertebral junction (Kamogawa et al. 2009). Since then, we have continued to develop this technique and have used it to visualize the lumbar (Misaki et al. 2009; Yamanaka et al. 2010; Kamogawa et al. 2012) and cervical (Kamogawa et al. 2014) nerve roots. The purpose of this study was to introduce virtual pathology of a cervical nerve root based on 3D MR/CT fusion images that show the bone and nerve root together on one color image that can be understood at a glance.

## Methods

### Subjects

We describe three patients who experienced neck or arm pain due to cervical radiculopathy. One patient had a cervical disc hernia and two had foraminal stenosis. Surgical treatment by posterior decompression under microscopy was performed in the two patients with foraminal stenosis.

CT images were obtained using an Asteion 4® 4-row CT unit (TOSHIBA, Tochigi, Japan) with the following parameters: tube voltage, 120 kV; tube current, 260 mA; slice thickness, 1 mm; rotational speed, 0.75 s/rotation; and slice thickness for reconstruction, 0.5 mm.

**Figure 1:**
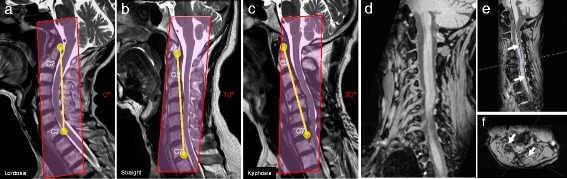
**Images showing the cutting angle for MR imaging and the mapping of the root on the workstation.** Images show a spine with cervical lordosis **(Panel a)**, a straight spine **(Panel b)**, and a spine with cervical kyphosis **(Panel c)**. The cervical root and ganglion are clearly visible on the double oblique view of the cervical spine **(d)**. The first oblique plane on the sagittal view **(Panel e, arrow)**, and the second oblique plane on the axial view through the right foramen **(Panel f, arrow)**.

MR images were obtained using an Echelon Vega® 1.5-T MR unit (HITACHI, Tokyo, Japan). Two MR imaging sequences: 3D myelography with 1.6-mm slice thickness (Balanced SG® and RSSG® by HITACHI) with following parameters: FOV, 250 mm, 267 mm; TR/TE, 9.8/4.9 ms, 21.0/9.9 ms; Flip angle, 45°, 10°; and slice thickness for reconstruction, 0.8 mm, 0.8 mm, respectively. The C5, C6, C7 and C8 roots were routinely imaged because they compose the brachial plexus. MR scanning was performed with one of three different cutting angles in the sagittal vertebral plane (Figure 1a, b, c). A line was placed from the top edge of the C2 vertebra to the cranio-dorsal corner of the C7 vertebra, and the cutting angle for MR scans was approximately 10° oblique clockwise from this line for patients with a straight neck, approximately 20° oblique clockwise from this line for patients with kyphosis, and at almost the same angle as this line for patients with lordosis (Figure 1a, b, c).

3D MR/CT fusion images were created using Synapse Vincent® computer workstation software (version 1 and version 3.3, FUJIFILM, Tokyo, Japan). 3D MR/CT fusion images were created using the following four steps, which are described in detail below: (1) Registration of the CT and MR data, (2) isolation of vertebral bodies on 3D CT images, (3) mapping of the spinal nerve roots on 3D MR images, and (4) final superposition.

#### Registration of the CT and MR data

The “superposition” application of the Synapse Vincent® workstation software was used to place the MR data onto the CT data. This tool is not exclusively designed for spinal use, and misalignment between the two modalities is possible. The gap was therefore manually corrected in the sagittal, coronal and axial planes. Special attention was paid to the alignment at the level of the affected nerve root, as indicated by clinical symptoms. Both CT and MR images should be obtained with the cervical spine in a neutral posture.

#### Isolation of vertebral bodies on 3D CT images

The “bony isolation” application of the Synapse Vincent® workstation software was used to automatically separate each vertebrae, from C1 to C7, using one click. Colors were then added to show each vertebral level more easily. Using this technique, the vertebrae can be made translucent at any density.

#### Mapping of the spinal nerve roots on 3D MR images

Two MR imaging sequences were used to obtain the spinal nerve data: Balanced SG® for cerebrospinal fluid and RSSG® for spinal cord, roots and disc.

Although it is easy to extract the spinal cord and cerebrospinal fluid from the imaging data, delicate skill is needed to extract the roots. Special attention must be paid to decide the angle for vertebral cutting in both MR operation and workstation processing. In workstation processing, the path of the root (Figure 1d) was identified using a double oblique cutting plane in sagittal and in axial on the vertebral (Figure 1e, f). These techniques were used to uncover the exact location of the root.

A high level of skill is required to map the path of the nerve root from the double oblique view (Figure 1d). The “center line editing” application of the Synapse Vincent® workstation software was used to place dots on the nerve roots in the multiplanar reconstruction image. Then, curved nerves roots were converted to straight nerve roots using the curved planar reformation method and the “center line editing” application of the Synapse Vincent® workstation software was used to identify the true long axis of the straight nerve. Next, the “contour editing” application of the workstation software was run 7–10 times to repair the edge of the straight nerve while rotating the root around the axis. Finally, the root was re-converted to the original shape. The resulting 3D nerve was placed on the multiplanar reconstruction screen and used like tracing paper to enable the user to manually fill in the dots to cut the unnecessary image near the root edge.

#### Final superposition

The 3D MR image was placed onto the 3D CT image, enabling the entire cervical spine to be observed. Each 3D fusion image required 2–3 h of processing at the workstation.

## Case description

### Case 1 (Figure 2)

**Figure 2:**
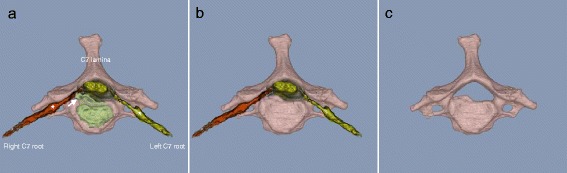
**3D MR/CT fusion images showing compression of the right C7 nerve root by an extruded herniated disc at the pre-foraminal site at C6/7.** Images show the C7 vertebra, C7 root and spinal root ganglion with **(Panel a)** and without **(Panel b)** the intervertebral disc and C7 vertebra alone **(Panel c)**. The affected side is shown in red. The disc is shown in green. Asterisks indicate the ganglion and the arrow indicates the herniated disc.

A 37-year-old man who was a towel designer had a 6-month history of right neck pain radiating to the right shoulder girdle with numbness of the right index finger and right middle finger due to a cervical herniated disc with C7 radiculopathy. Recently, he had experienced sleep disturbance and had to sit up to sleep for 3 days because of severe pain. The patient underwent conservative treatment for 3 months and recovered with no pain (Figure 2).

### Case 2 (Figure 3)

**Figure 3:**
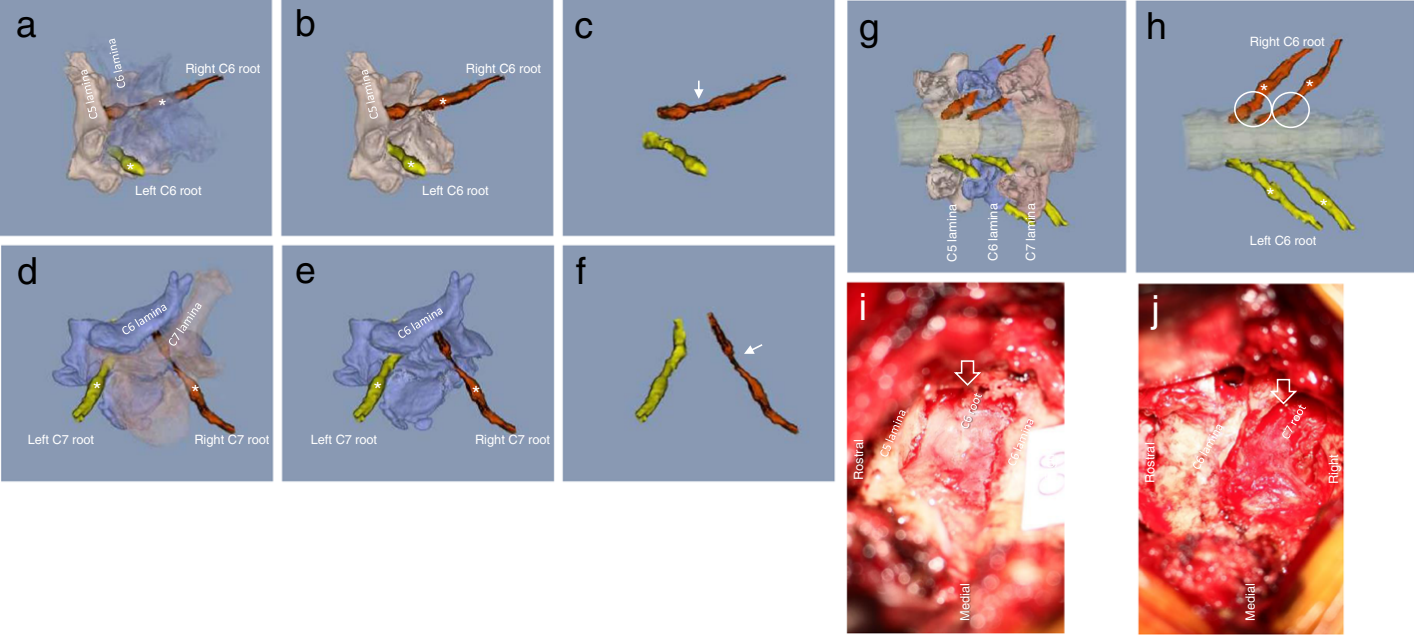
**3D MR/CT fusion images showing compression of the right C6 and C7 nerve roots at the entrance of the foramen due to foraminal encroachment.** Light pink indicates the C5 vertebra and blue indicates the C6 vertebra. The affected roots are shown in red. Asterisks indicate ganglion. The posterolateral-inferior view of the affected root is the angle that most clearly show the flattened portion of the root. Images are shown with semi-translucent vertebrae **(Panel a and d)**, with only one of the vertebrae **(Panel b and e)**, and with neither of the vertebrae **(Panel c and f)**. Note that the observed root direction is different for the C6 and the C7 root. Arrows indicate the flattening of the affected root. In a simulated view of the operation, the unnecessary bony information was removed from the image until each pedicle and affected root was visible **(Panel g)**. The pedicle is the important bony element for partial pediclectomy during microsurgical decompression. Panel **i** (C6 root) and **j** (C7 root) show intra-operative microscopic images from the areas circled on panel **h** that shows the posterior-anterior view of the flattened root. In panels **i** and **j** the compressed root is red and flattened with adhesion (open arrows). Note that we met the wide face of the affected root in operation but not the narrow face. About 1 cm of the root was visible during the operation and confirmed the accuracy of the fusion image.

A 67-year-old man who was a tanker building designer had degenerative cervical spondylosis with both C6 and C7 radiculopathy. He was a heavy smoker. The main clinical findings were a 4.5-year history of severe neck and right arm pain. The patient received several conservative treatments, including modified satellite ganglion block 61 times. Selective right C6 and C7 root block caused reproducible pain and reduced the pain level. Marked radicular pain of the right C6 and C7 nerve root was confirmed by both root block and 3D MR/CT fusion imaging. The patient underwent cervical foraminotomy (both C6 and C7) under microscopy. After surgery, radicular pain completely disappeared but numbness of the finger partially remained. The patient recovered with only slight neck stiffness as a residual symptom. This case was included in our previous report on root groove view (Kamogawa et al. 2014), but we show the pathoimagiology of the root for first time in this report (Figure 3).

### Case 3 (Figure 4)

**Figure 4:**
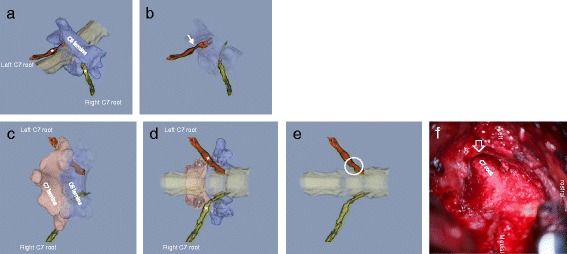
**3D MR/CT fusion image showing compression of the left C7 nerve root by foraminal stenosis.** Blue indicates the C6 vertebra and pink indicates the C7 vertebra. The affected roots are shown in red. Asterisks indicate ganglion. The posterolateral-inferior view of the affected root showed the flattening of the root most clearly at pre-ganglion site **(Panel a)**, with semi-translucent C6 vertebra **(Panel b)**. A simulated view of the operation reveals the relation between the affected C7 root and the superior articular process of C7 vertebra **(Panel c)** and removal of unnecessary lamina from the image reveals the pedicle and the affected root **(Panel d)**. Panel f shows an intra-operative microscopic image from the area circled on panel **e**. The intra-operative pathology evident in panel **f** (open arrow indicates C7 root) is almost the same as that evident in the fusion image. Note that panel **e** shows the posterior-anterior view of the flattened root and panel **b** shows almost the lateral view. The surgeon must be aware of the pathoanatomical condition of a compressed root with a tortuous or twisted appearance.

A 42-year-old man who was a desk worker had degenerative cervical spondylosis with C7 radiculopathy. He had neck pain, left shoulder stiffness and radicular pain of left arm and had experienced sleep disturbance for 6 years. He was medicated with non-steroidal anti-inflammatory drugs. In the 2 years prior to surgery the patient had received modified satellite ganglion block 10 times, but had progressively worsening symptoms. Selective C7 root block was performed for both diagnosis and treatment and caused reproducible pain and reduced the pain level. The patient underwent cervical foraminotomy (C7) under microscopy under the diagnosis made both by root block and 3D MR/CT fusion imaging. After surgery, he returned to health and the pain disappeared (Figure 4).

## Discussion

For numerous reasons (Kamogawa et al. 2012), the pathology of cervical radiculopathy is difficult to identify using routine two-dimensional MR images in the axial and sagittal plane. This is further compounded by the fact that the area of the cervical spine that is composed of the root and intervertebral foramen is hidden on two-dimensional MR images (Kamogawa et al. 2014). 3D MR imaging has also failed to provide detailed images of the cervical root due to poor methodological quality. The 3D MR/CT fusion imaging technique enhances visualization of pathoanatomy in this hidden root area. Although the nerve roots are located in the upper part of the foramen at the lumbar level, they occupy the lower part of the foramen at the cervical level (Diano et al. 2013), and the upper portion of the foramen contains blood vessels and fat. The paucity of fat in the cervical region and the smaller size of the foramina make the cervical roots more difficult to visualize than the lumbar roots (Pech et al. 1985). Moreover, the cervical roots are small and soft and can change shape during motion. Characteristic anatomical features of the cervical nerve roots include curved running, no merkmal, and no enhancement with contrast media. We have been trying to develop a method that can identify the border between the nerve root and the surrounding connective tissue, that is, between water and fat. Our 3D MR/CT fusion imaging technique provides two distinct advantages for diagnosis of cervical radiculopathy. First, the isolation of individual vertebra clarifies the deformities of the whole root groove (Ebraheim et al. 1996), including both the uncinate process and superior articular process. Second, the whole root can be visualized.

Uncovertebral osteophytes are the most common cause of nerve root compression in cervical spondylosis (Lyon E 1945; Raynor RB 1983; Lu et al. 1998; Bozbuğa et al. 1999; Uğur et al. 2000; Civelek et al. 2007; Hartman J 2014). Further narrowing of the intervertebral foramen can occur due to posterior encroachment secondary to zygapophysial joint degeneration, ligament flava, or periradicular fibrous tissue thickening and further anterior encroachment can occur due to a protruding disc or a bulging posterior longitudinal ligament (Cave et al. 1955; Tanaka et al. 2000; Shen et al. 2004). Pech et al. reported the appearance of the cervical neural foramina and contents on CT images in detail and pointed out that the dorsal nerve roots and ganglion contacted the superior articular process and the ventral nerve roots contacted the uncinate process (Pech et al. 1985).

MR images can distinguish the dorsal and ventral nerve roots under optimal conditions, but cannot do so in the majority of patients. 3D depiction of nerve roots can lead to beautiful and detailed images. In our previous study of cervical MR imaging of about 400 patients we found that root image clarity was excellent in 30%, good in 37%, moderate in 23%, poor in 3%, and impossible to evaluate in 7% (unpublished data). In the current study, we only used 3D MR/CT fusion images that depicted the nerve root with excellent or good clarity. From this point of view, we cannot emphasize enough that clear MR/CT fusion images require a skillful MR operating technique and a high level of skill from the radiologist when making the images by workstation. From our experience, clear MR images can be obtained from patients who show bradycardia and autonomic imbalance and who remain immobile and calm when surrounded by loud MRI sounds.

On the two-dimensional images created from the 3D MR/CT fusion image, the flattened portion of the root appeared wide from some directions and narrow from other directions. During posterior decompression surgery performed under microscopy, the surgeon might meet the wide face of the affected root, but not the narrow face. The surgeon can identify the narrowest face of the root if they view the MR/CT fusion image from the posterolateral-inferior direction. Surgeons use MR/CT fusion images as a pre-operative map and for intraoperative navigation. The MR/CT fusion images can also be used as educational materials for all hospital staff and for patients and patients’ families who provide informed consent for treatments.

To our knowledge, this is the first study to reveal the impingement and flattening of cervical nerve roots along with bone deformities in 3D on one image. There is no literature describing the benefits of these images of the cervical spine and, consequently, there is lack of awareness of their potential benefit in interpreting the pathology of the roots and surrounding environment and a lack of awareness of how to obtain high-quality 3D MR imaging data. In this report, we revealed how to visualize the cervical nerve roots in 3D. Patients that have experienced pain for a long duration might exhibit a flattening or constriction of the affected root at a pre-ganglion site. 3D MR/CT fusion images allow more accurate assessment of foraminal pathology and thus more appropriately directed treatment than 2D MR or CT images.

We propose the term “pathoimagiology” to refer to the combination of pathology and imaging. Spondylotic cervical radiculopathy is not malignant, therefore we were not able to take samples to examine histopathology. Moreover even when decompression surgery is performed, the surgeon rarely exposed the ganglion in the cervical spine because it is located far lateral to the decompression site. Therefore, surgeons need new way to evaluate the root shape.

In future studies we will try to classify the cervical compressed root according to its deformity or the severity of the bony spur assessed using both pre-operative images and intra-operative pathology. In addition, we will use 3D MR/CT fusion imaging to image trauma or tumors in the cervical spine and brachial plexus.

Moreover, we will refine the current technique to increase the clarity of the spinal root ganglia, thus enabling surgeons to qualitatively evaluate the pathology (Yamashita et al. 2009; Eguchi et al. 2011) (e.g., edema, demyelination, or axonal injury) as well as shape of the root. We will investigate the relation between the virtual pathology and clinical symptoms or the results of electro-diagnostic studies such as needle electromyography and nerve-conduction tests. In this study, the nerve root mapping depended on manual input and development of an automatic mapping technique is underway. We believe that automated techniques will be able to detect borders that human operators cannot see with the naked eye.

Although our technique is associated with some problems such as misalignment between the two imaging modalities, poor convenience, and applicability, we intend to continue with development to further improve this fusion imaging technique.

### Ethical standards and patient consent

We declare that all human and animal studies have been approved by the Japanese Orthopaedic Association and have therefore been performed in accordance with the ethical standards laid down in the 1964 Declaration of Helsinki and its later amendments. We declare that all patients gave informed consent prior to inclusion in this study.
